# Effect of vitamin D status on adult COVID-19 pneumonia induced by Delta variant: A longitudinal, real-world cohort study

**DOI:** 10.3389/fmed.2023.1121256

**Published:** 2023-03-24

**Authors:** Hua Huang, Jiawei Zheng, Yan Liu, Qunhe Zhou, Denggao Peng

**Affiliations:** ^1^Department of Radiology, Shenzhen Third People’s Hospital, Second Hospital Affiliated to Southern University of Science and Technology, Shenzhen, China; ^2^Department of Emergency Medicine, Shenzhen Third People’s Hospital, Second Hospital Affiliated to Southern University of Science and Technology, Shenzhen, China; ^3^Department of General Practice, Shenzhen Third People’s Hospital, Second Hospital Affiliated to Southern University of Science and Technology, Shenzhen, China

**Keywords:** artificial intelligence, Delta, pneumonia, variant, vitamin D

## Abstract

**Objective:**

The effect of vitamin D status on adult COVID-19 pneumonia induced by Delta variant remains to be further explored.

**Methods:**

A longitudinal, real-world cohort study was performed. Artificial intelligence (AI) was used to identify and measure pneumonia lesions. All cases with pneumonia were divided into the vitamin D deficiency (VDD) and control groups according to serum 25-hydroxyvitamin D concentration. Lesion dynamics were observed within six time periods after the onset of pneumonia.

**Results:**

A total of 161 cases were included, of which 101 (63%) were male and 46 (29%) presented with pneumonia. The median age and baseline 25-hydroxyvitamin D concentrations were 37 years and 21 ng/ml, respectively. Age, fibrinogen, and SARS-CoV-2 IgG titer on admission were independent predictors for the onset of pneumonia. After the onset of pneumonia, patients in the VDD group (*n* = 18) had higher percentage of fever (33 vs. 7.1%; *p* = 0.04) than those in the control group (*n* = 28); the interval of pneumonia resolution was longer (28 vs. 21 days; *p* = 0.02); lesions progressed more rapidly (*p* = 0.01) within 3 to 7 days and improved more slowly (*p* = 0.007) within more than 28 days; notably, simultaneous interleukin-6 (18.7 vs. 14.6 pg/ml; *p* = 0.04) levels were higher, and cycle thresholds for N gene (22.8 vs. 31.3; *p* = 0.04) and ORF1ab gene (20.9 vs. 28.7; *p* = 0.03) were lower within 3 to 7 days.

**Conclusion:**

Vitamin D status may have effects on the progression and resolution, but not the onset of Delta variant-induced pneumonia in adults. Computed tomography image diagnosis system based on AI may have promising applications in the surveillance and diagnosis of novel SARS-CoV-2 variant-induced pneumonia.

## Introduction

The uncertainty and unpredictability of coronavirus disease-2019 (COVID-19) epidemic caused by severe acute respiratory syndrome coronavirus-2 (SARS-CoV-2) have made it difficult for governments and health service systems to make timely decisions and take appropriate prevention and control measures, which has become a major global public health challenge (1). With the emergence of novel variants such as Delta and Omicron, SARS-CoV-2 is becoming more transmissible, less pathogenic and lung-invasive, significantly different from the ancestral virus (2). However, COVID-19 pneumonia remains the leading cause of severe illness and death, especially among older individuals with co-morbidities. Computed tomography (CT) imaging features high spatial resolution, is not susceptible to interference from structures outside the plane, and can display the details of lesions in multiple planes and directions. Therefore, chest CT plays an important role in the diagnosis of COVID-19 pneumonia and has important significance in the stratification of severity, drug efficacy evaluation, and prognosis (3). Notably, the characteristics and dynamics of pneumonia lesions induced by different novel variants are not well defined and inconclusive because most of them are mild, difficult to quantitative measure, and not easily distinguishable from other viral pneumonia (4, 5).

Vitamin D comes in two forms: ergocalciferol (D_2_) and cholecalciferol (D_3_). The former is a steroid found in some plants, but mainly in fungi. The latter is synthesized by solar ultraviolet-B irradiation in animal skin (6). As a fat-soluble prohormone, vitamin D is sequentially hydroxylated at C25 and C1 to produce the 25-hydroxyvitamin D [25(OH)D] in the liver with further metabolism in the kidney to the biologically active 1,25-dihydroxyvitamin D [1,25(OH)_2_D] (7). In addition to maintaining the homeostasis of calcium and phosphorus, vitamin D has immunomodulatory properties by stimulating the expression of immune function receptors in airway epithelial cells to comprehensively prevent acute respiratory infections (8–10). More importantly, vitamin D may modulate the shift of immune response toward T helper 1 (Th1) or Th2. Typically, the Th1-type immune response cascades to release pro-inflammatory cytokines, which may thereby cause cytokine storm and induce acute lung injury (11). In contrast, the Th2-type immune response has anti-inflammatory effects by activating B cell maturation and, in turn, producing pathogen-specific antibodies (12). In conditions of vitamin D deficiency (VDD) or insufficiency, the body’s immune system shifts toward the Th1 direction. Multiple studies have suggested that vitamin D may reduce COVID-19 mortality and severity in hospitalized patients, especially in adults with VDD (13, 14). Our previous clinical study found also higher viral loads and larger pneumonia lesions in vitamin D-insufficient children infected with Omicron BA.2, suggesting that vitamin D status may be involved in the pathogenesis of Omicron variant-induced pneumonia (15). However, the CT imaging dynamics of pneumonia induced by Delta variant and the specific effect of vitamin D status on it have not been currently reported in the literature. This longitudinal, real-world cohort study may be necessary for the prevention and treatment of COVID-19 pneumonia.

## Materials and methods

### Study design and data collection

The institutional COVID-19 database of Shenzhen Third People’s Hospital was used, which was continuously updated until April 1, 2022. Our first case of Delta variant infection was confirmed on June 14, 2021. A longitudinal, real-world cohort study was conducted to evaluate the effect of vitamin D status on adult COVID-19 pneumonia induced by the Delta variant. Prior to the formal analysis, our research team discussed and developed case selection criteria and data collection tables internally, and conducted training for each member to ensure data quality. Two authors (JZ and QZ) independently retrieved electronic medical records and identified eligible cases, and a third author (YL) judged and ruled in the cases that consensus was not reached. The authors (HH and DP) participating in the final data analysis were blinded to the grouping settings and conditions.

Inclusion criteria:

1. From June 14, 2021 to April 1, 2022, all adult patients infected with the SARS-CoV-2 Delta variant who were tested for serum 25(OH)D concentration on admission and continuously monitored for chest CT during hospitalization and after discharge.

Exclusion criteria:

1. Cases with missing key data or lost to follow-up.
2. Cases with pre-existing chronic structural lung disease, including but not limited to chronic obstructive pulmonary disease (COPD), bronchiectasis, tuberculosis, lung tumors, or pre-existing immunosuppression.

### Clinical definition and classification

Adult were defined as being more than 18 years old. All COVID-19 patients were admitted to Shenzhen Third People’s Hospital for isolation and treatment, underwent at least one bi-weekly follow-up after discharge. Relevant examinations were completed as routine procedures. Quantitative reverse transcriptase-polymerase chain reaction (qRT-PCR) was used to detect SARS-CoV-2 positive and viral load in nasopharyngeal swab samples. A cycle threshold of <40 for N gene or ORF1ab gene was defined as positive. Whole-genome sequencing and bioinformatics analysis were used to confirm SARS-CoV-2 variant types. Fever was recognized when body temperature is higher than or equal to 37.3°C. Respiratory symptoms included nasal congestion, runny nose, sneezing, sore throat, cough, expectoration, chest pain, and dyspnea. Vaccination status was classified as unvaccinated, routinely vaccinated and booster vaccinated. The routinely vaccinated was defined as vaccination with a standard two-dose inactivated vaccine. The booster vaccinated was defined as vaccination with an additional third dose of homologous inactivated vaccine.

### Imaging evaluation

Non-contrast thin-section chest CT was performed using Shanghai uCT760 64-row spiral CT machine (reconstruction slice thickness 0.625 mm). All image data were observed within the lung window with a window settings (width 1,600 HU; level −550 HU). COVID-19 pneumonia lesions on chest CT including ground-glass opacities (GGOs), consolidation, and nodular opacities were automatically identified and quantified using artificial intelligence (AI) software (InferRead CT Pneumonia, V1.1.3.0, Tuixiang, Beijing, China). The normalized and individualized lesion volume (the ratio of total lesion volume to simultaneous total lung volume) was calculated using the same method as in our previous study (15). The overwhelming absorption of the lesion (greater than 95% of the maximum normalized lesion volume) was defined as the resolution of pneumonia. All patients with pneumonia underwent chest CT every 3 days during hospitalization and once a week after discharge until resolution. Given that the median (interquartile range, IQR) length of hospital stay and interval of pneumonia resolution were 24 (18–29) and 24 (14.5–30.5) days, respectively, we selected six time periods from T_1_ to T_6_ after the onset of pneumonia to observe lesion dynamics. T_1_ to T_6_ represent the following: T_1_ ≤ 3; 3 < T_2_ ≤ 7; 7 < T_3_ ≤ 14; 14 < T_4_ ≤ 21; 21 < T_5_ ≤ 28; T_6_ > 28 days. Meanwhile, the ratio of normalized lesion volume in T_x + 1_ to T_x_ was calculated to observe the longitudinal changes of lesions. The T_x + 1_ to T_x_ ratio greater than or less than 1 indicates progression or improvement, respectively. If there were two or more measurements in the same time period, their average was calculated.

### Laboratory parameter

Serum 25(OH)D concentrations were detected using vitamin D Total Assay Kit (ADVIA Centaur®, Siemens Medical Diagnostics Inc., New York 10,591, United States) based on chemiluminescence method. Vitamin D status (deficient or not) was classified into VDD and non-VDD (control) based on baseline 25(OH)D levels, with reference to cut-offs commonly used in Global Consensus Recommendations: (1) VDD was defined as serum 25(OH)D concentration < 20 ng/ml; (2) Control was defined as serum 25(OH)D concentration ≥ 20 ng/ml (16).

The specific IgM and IgG antibodies against SARS-CoV-2 in serum specimens were quantitatively determined using the Novel Coronavirus (2019-nCoV) Antibody Detection Kit based on chemiluminescence method. The observed IgM titers ≥1 AU/ml or IgG titers ≥10 AU/ml were considered positive, respectively. Laboratory results in the first day after admission (T_0_) and simultaneous time periods from T_1_ to T_5_ after the onset of COVID-19 pneumonia were observed.

### Statistical analysis

All analyses were conducted by using of IBM Statistical Product and Service Solutions Version 26 (SPSS 26.0, IBM Inc., Chicago, IL) and GraphPad Prism 8 software. Descriptive statistics were summarized as median [IQR] or [mean ± standard deviation (SD)] for continuous variables, depending on whether their distributions are normal or not, and frequencies and percentages for categorical variables. Parametric tests (independent sample *t*-test) or non-parametric tests (Mann–Whitney U-test) for continuous variables, and Pearson Chi-square test or Fisher exact test for categorical variables were used. Variables with *p* < 0.1 in the univariate comparison results were entered into a multivariate binary logistic model. Model fitness was assessed with the Hosmer–Lemeshow goodness-of-fit test. *p* < 0.05 was considered as statistically significant in all tests if applied.

## Results

### Demographic and clinical characteristics and baseline laboratory results

Till April 1, 2022, a total of 161 discharged adult patients infected with the Delta variant were included in this study, of which 101 (63%) were male and 46 (29%) presented with pneumonia. The median (IQR) age and baseline 25-hydroxyvitamin D concentration were 37 (28–47) years and 21 (17–27) ng/ml, respectively. There were significant differences in age (34 vs. 43 years; *p* < 0.001), SARS-CoV-2 IgM titer (0.3 vs. 0.1 AU/ml; *p* = 0.002), SARS-CoV-2 IgG seropositive (84 vs. 44%; *p* < 0.001), and titer (82.8 vs. 3.3 AU/ml; *p* < 0.001), lymphocyte (1.36 vs. 0.87 × 10^9/L; *p* = 0.002) and platelet count (224 vs. 200 × 10^9/L; *p* = 0.04), fibrinogen (2.9 vs. 3.3 g/L; *p* < 0.001), and aspartate aminotransferase (27 vs. 29 U/L; *p* = 0.01) between the non-pneumonia (*n* = 115) and pneumonia (*n* = 46) groups. Detailed demographic and clinical characteristics and baseline laboratory parameter are presented in Table 1.

**Table 1:** Univariate comparison between the non-pneumonia and pneumonia groups on admission.

Univariates	Non-pneumonia (*n* = 115)	Pneumonia (*n* = 46)	*p* value
Age (years), Median (IQR)	34 (26–43)	43 (35–55)	<0.001
Male gender, *n* (%)	71 (62%)	30 (65%)	0.68
Vitamin D status (deficiency)	52 (45%)	18 (39%)	0.48
Serum 25(OH)D concentration (ng/ml)	21 (17–25)	22 (17–29)	0.28
Vaccination status			0.99
Unvaccinated	52 (45%)	21 (46%)	
Routinely vaccinated	50 (44%)	20 (43%)	
Booster vaccinated	13 (11%)	5 (11%)	
SARS-CoV-2 IgM seropositive	8 (7.0%)	4 (8.7%)	0.74
SARS-CoV-2 IgM titer (AU/ml)	0.3 (0.2–0.8)	0.1 (0.07–0.4)	0.002
SARS-CoV-2 IgG seropositive	96 (84%)	20 (44%)	<0.001
SARS-CoV-2 IgG titer (AU/ml)	82.8 (10.4–218.7)	3.3 (0.6–21.7)	<0.001
Comorbidities			
Hypertension	4 (3.5%)	4 (8.7%)	0.23
Diabetes	0 (0%)	3 (6.5%)	0.02
White blood cell count (×10^9/L; 3.5–9.5)	6.4 (5.1–7.7)	6.5 (4.6–8.9)	0.97
Neutrophil count (×10^9/L; 1.8–6.3)	4.2 (3.4–5.6)	4.8 (3.4–7.2)	0.33
Lymphocyte count (×10^9/L; 1.1–3.2)	1.4 (0.9–1.7)	0.9 (0.7–1.3)	0.002
Platelet count (×10^9/L; 125–350)	224 (196–252)	200 (167–237)	0.04
ESR (mm/h; 0–20)	5 (3–12)	6 (3–16)	0.25
hsCRP (mg/L; 0–8)	1.0 (0.4–3.1)	1.7 (0.4–4.1)	0.76
Procalcitonin (ng/ml; 0–0.1)	0.05 (0.04–0.07)	0.05 (0.04–0.07)	0.94
IL-6 (pg/ml; 0–7)	12.7 (9.4–18.5)	13.5 (10.7–18.2)	0.55
PT (Sec; 11–15.1)	13.3 (12.9–13.9)	13.2 (12.9–13.7)	0.81
APTT (Sec; 28–43.5)	38.2 (35.6–41.4)	38.5 (37.2–41.7)	0.22
Fibrinogen (g/L; 2–4)	2.9 (2.4–3.4)	3.3 (2.9–3.9)	<0.001
D-dimer (μg/ml; 0–0.5)	0.2 (0.2–0.3)	0.3 (0.2–0.3)	0.41
Alanine aminotransferase (U/L; 0–45)	28 (22–43)	32 (23–42)	0.43
Aspartate aminotransferase (U/L; 0–45)	27 (23–32)	29 (25–36)	0.01
Creatinine (μmol/L; 41–91)	68 (53–82)	62 (56–71)	0.36
Troponin I (μg/L; 0–0.034)	0.02 (0.01–0.02)	0.01 (0.01–0.02)	0.28
Lactic acid (mmol/L; 0.5–1.5)	1.4 (1.1–1.9)	1.4 (1.0–1.8)	0.48
PaO_2_/FiO_2_ ratio (mmHg; 400–500)	450 (413–498)	425 (394–490)	0.06
Cycle threshold for N gene	28.7 (19.2–34.9)	23.5 (18.8–34.1)	0.35
Cycle threshold for ORF1ab gene	26.3 (18.1–33.7)	22.4 (18.6–29.9)	0.21

Abbreviations: 25(OH)D, 25 hydroxyvitamin D; APTT, activated partial thromboplastin time; ESR, erythrocyte sedimentation rate; FiO2, fraction of inspired oxygen; hsCRP, high-sensitivity C-reactive protein; IL-6, interleukin-6; IQR, interquartile range; PaO2, arterial oxygen partial pressure; PT, prothrombin time; SARS-CoV-2, severe acute respiratory syndrome coronavirus-2.

### Independent predictors for the onset of COVID-19 pneumonia

Variables with *p* < 0.1 including age, SARS-CoV-2 IgM titer, SARS-CoV-2 IgG titer, lymphocyte and platelet count, fibrinogen, aspartate aminotransferase, diabetes comorbidity, and ratio of arterial oxygen partial pressure (PaO_2_) to fraction of inspired oxygen (FiO_2_) were entered into a backward stepwise multivariate binary logistic regression model. The last step was to obtain three independent predictors of age, fibrinogen, and SARS-CoV-2 IgG titer (Table 2). Goodness-of-fit testing was used to assess deviations between observed and expected values. A *p* value of >0.05 implies no significant difference. Here, the *p* value of Hosmer–Lemeshow test in our model was 0.69.

**Table 2:** Independent predictors for the onset of Delta variant-induced pneumonia on admission.

Univariates	B	SE	Wald	*p* value	OR	95%CI
Age	0.055	0.018	9.3	0.002	1.06	1.02–1.09
Fibrinogen	0.856	0.255	11.3	0.001	2.35	1.43–3.88
SARS-CoV-2 IgG titer	−0.005	0.002	6.3	0.01	0.995	0.991–0.999

Abbreviations: CI, confidence interval; OR, odds ratio; SARS-CoV-2, severe acute respiratory syndrome coronavirus-2; SE, standard error.

### Effects of vitamin D status on COVID-19 pneumonia

**Figure 1:**
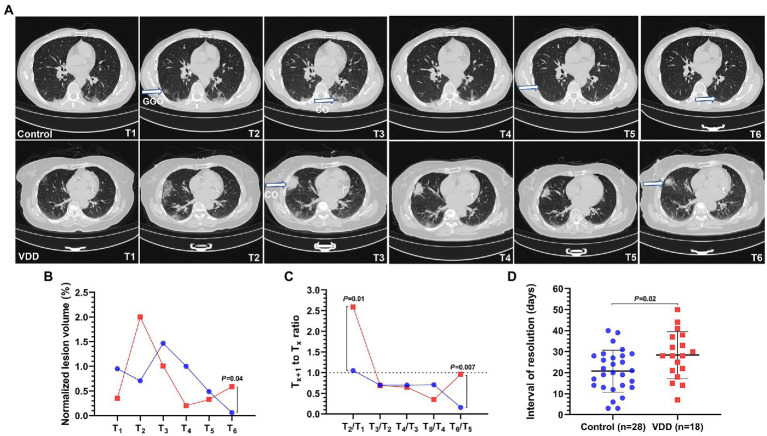
Lesion dynamics in six time periods from T_1_ to T_6_ after the onset of COVID-19 pneumonia induced by the Delta variant. T_1_ to T_6_ represent the following: T_1_ ≤ 3; 3 < T_2_ ≤ 7; 7 < T_3_ ≤ 14; 14 < T_4_ ≤ 21; 21 < T_5_ ≤ 28; T_6_ > 28 days. Pneumonia lesions on computed tomography (CT) were identified and measured by artificial intelligence (AI) and radiologists. The blue or red connecting line and symbols represent the control or VDD group, *n* = 7–28 or *n* = 9–18 per condition, respectively. Symbols in the line chart represent median. Scatter dot plot and error bars represent mean and standard deviation. GGO, ground-glass opacity; VDD, vitamin D deficiency; Control, without VDD. **(A)** COVID-19 pneumonia lesions mainly included GGO and consolidation (CO). **(B)** The normalized lesion volume between the control and VDD cases was significantly different (0.6 vs. 0.06%) within more than 28 days. **(C)** The T_x + 1_ to T_x_ ratio of normalized lesion volume greater than or less than 1 indicates progression or improvement, respectively. Lesions in the VDD group progressed more rapidly (2.6 vs. 1.1) within 3 to 7 days and improved more slowly (0.96 vs. 0.16) after 28 days than those in the control group. **(D)** The interval of pneumonia resolution in the VDD group was longer (28 vs. 21 days) than that in the control group.

All cases with pneumonia were divided into the control (*n* = 28) and VDD (*n* = 18) groups. The average age were 48 and 41 years, respectively. Differences in percentage of male gender, vaccination status, and comorbidities were not statistically significant. Two (7.1%) cases in the control group and six (33%) cases in the VDD group presented with fever, with significant difference (*p* = 0.04); 17 (61%) and 14 (78%) presented with respiratory symptoms. The median (IQR) interval of negative RNA conversion and interval from admission to pneumonia onset were 19 (14–26) days and 3 (0.3–5) days, respectively. There was no statistical difference between the two groups (Table 3). The lesions of Delta variant-induced pneumonia mainly included GGOs and consolidation (Figure 1A). After the onset of pneumonia, the VDD group had larger normalized lesion volume (0.6 vs. 0.06%; *p* = 0.04) within more than 28 days (Figure 1B; Supplementary Table S1); more rapidly progressed lesions (2.6 vs. 1.1; *p* = 0.01) within 3 to 7 days and slowly improved lesions (0.96 vs. 0.16; *p* = 0.007) within more than 28 days (Figure 1C); longer interval of pneumonia resolution (Figure 1D) compared with the control group (Supplementary Table S2).

**Table 3:** Demographic and clinical characteristics of patients with pneumonia induced by the Delta variant.

Univariates	Control (*n* = 28)	VDD (*n* = 18)	*p* value
Age (years), Mean ± SD	48 ± 12	41 ± 14	0.08
Male gender, *n* (%)	20 (71%)	10 (56%)	0.27
Vaccination status			0.42
Unvaccinated	12 (43%)	9 (50%)	
Routinely vaccinated	14 (50%)	6 (33%)	
Booster vaccinated	2 (7%)	3 (17%)	
*Comorbidities*			
Hypertension	3 (11%)	1 (5.6%)	1.00
Diabetes	2 (7.1%)	1 (5.6%)	1.00
SARS-CoV-2 IgM seropositive	3 (11%)	1 (5.6%)	1.00
SARS-CoV-2 IgG seropositive	12 (43%)	8 (44%)	0.92
Fever	2 (7.1%)	6 (33%)	0.04
Duration of fever (days)	8 ± 2	5 ± 2	0.15
Peak body temperature (°C)	39 ± 0.3	38.1 ± 0.7	0.16
Respiratory symptoms	17 (61%)	14 (78%)	0.23
Duration of respiratory symptoms (days)	23 ± 13	25 ± 13	0.57
Length of hospital stay (days), Median (IQR)	25.5 (18–30)	23.5 (20–26)	0.54
Interval of negative nucleic acid conversion (days)	17 (14–26.5)	19 (17–24)	0.73
Interval from admission to pneumonia onset (days)	3 (0.5–5.5)	3 (0–5)	0.59

Abbreviations: IQR, interquartile range; SD, standard deviation; SARS-CoV-2, severe acute respiratory syndrome coronavirus-2; VDD, vitamin D deficiency.

### Simultaneous laboratory result after the onset of pneumonia

**Figure 2:**
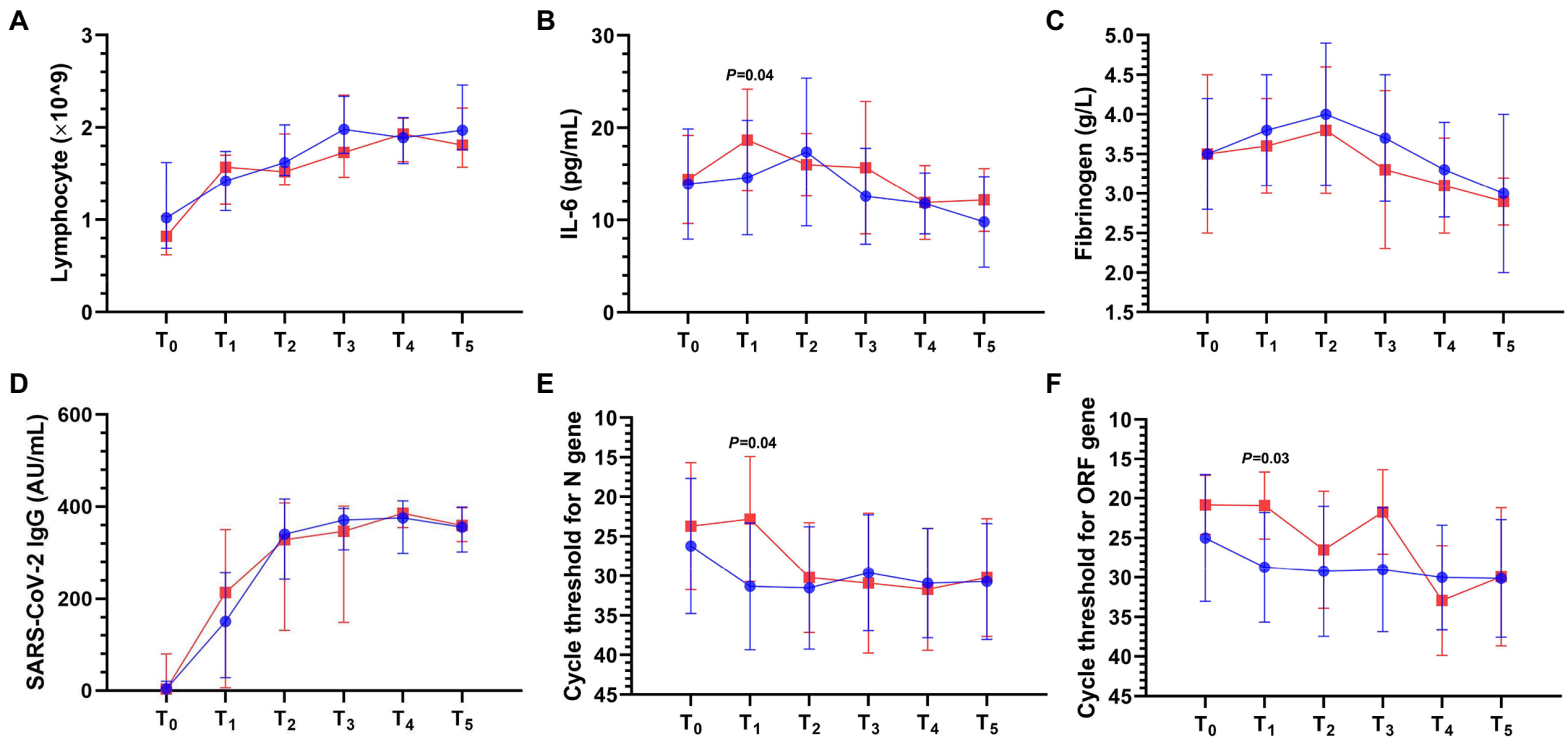
Laboratory result dynamics in the first day after admission (T_0_) and simultaneous time periods from T_1_ to T_5_ after the onset of COVID-19 pneumonia. T_1_ to T_5_ represent the following: T_1_ ≤ 3; 3 < T_2_ ≤ 7; 7 < T_3_ ≤ 14; 14 < T_4_ ≤ 21; 21 < T_5_ ≤ 28 days. IL-6, interleukin-6; SARS-CoV-2, severe acute respiratory syndrome coronavirus-2. The blue or red connecting line and symbols represent the control or VDD group, *n* = 8–28 or *n* = 5–18 per condition, respectively. Symbols and error bars in the line chart represent median and interquartile range **(A,D)** or mean and standard deviation **(B,C,E,F)**. **(A)** Lymphocyte. **(B)** IL-6 in the VDD group was higher (18.7 ± 5.5 vs. 14.6 ± 6.2 pg/ml) than that in the control group within 3 days. **(C)** Fibrinogen. **(D)** SARS-CoV-2 IgG titer. **(E)** Cycle threshold for N gene in the VDD group was lower (22.8 ± 7.9 vs. 31.3 ± 8) than that in the control group within 3 days. **(F)** Cycle threshold for ORF1ab gene in the VDD group was lower (20.9 ± 4.2 vs. 28.7 ± 6.9) than that in the control group within 3 days.

Compared with the control group, the VDD group had higher interleukin-6 (IL-6; 18.7 ± 5.5 vs. 14.6 ± 6.2 pg/ml; *p* = 0.04; Figure 2B) and lower cycle thresholds for N gene (22.8 ± 7.9 vs. 31.3 ± 8; *p* = 0.04; Figure 2E) and ORF1ab gene (20.9 ± 4.2 vs. 28.7 ± 6.9; *p* = 0.03; Figure 2F) within 3 days. There was no statistical difference in lymphocyte count (Figure 2A), fibrinogen (Figure 2C), D-dimer, SARS-CoV-2 IgM titer, SARS-CoV-2 IgG titer (Figure 2D), and procalcitonin between the two groups (Supplementary Table S3).

## Discussion

To our knowledge, this is the first longitudinal, real-world cohort study on the effect of vitamin D status on Delta variant-induced COVID-19 pneumonia in adults so far. It is well known that there have been multiple outbreaks of SARS-CoV-2 variants around the world, including Alpha, Beta, Gamma, Delta, and Omicron in chronological order. Among these outbreaks, the incidence of viral pneumonia decreased significantly with the chronological order of the variant types (17). Moreover, the clinical and CT imaging features of pneumonia induced by the novel variant were significantly different from those by the wild-type (WT) strain and preceding variants (5, 18). Currently, the Omicron variant and its novel sublineages, such as BA.2.12.1, BA.4/BA.5, and BF.7, quickly took over Delta and became the overwhelmingly dominant strain worldwide. The application of clinical and CT imaging knowledge based on WT to guide the evaluation and treatment of Omicron variant-induced pneumonia may lead to certain bias. Furthermore, several recent cross-sectional studies have demonstrated that the Omicron variant shows fewer, less severe, and more “atypical” lesions on chest CT than the Delta variant (5, 18, 19). Despite differences and inconsistencies, Delta is still likely to be closest to Omicron in terms of timing of occurrence, vaccination status, and clinical and imaging features. Our longitudinal observational study clearly described the CT imaging dynamics of Delta variant-induced pneumonia by innovatively combining AI with radiologists to identify and measure lesions, and highlighted the effects of vitamin D status on lesion dynamics and simultaneous laboratory results, which could provide references for the clinical management of Omicron variant-induced pneumonia.

Some studies have reported that older age, comorbidities, coagulation dysfunction, and unvaccinated status may increase the risk of COVID-19-related acute respiratory distress syndrome (ARDS) and pneumonia (14, 20–22). Our multivariate binary logistic regression analysis obtained three independent predictors of age, fibrinogen, and SARS-CoV-2 IgG titer. The median age of patients with pneumonia was significantly higher than that of patients without pneumonia (43 vs. 34 years). Age-related comorbidities are hypothesized to be important contributing factors. However, there was no statistical difference in the hypertension comorbidity between the two groups, which may be related to the higher prevalence of Delta infection in younger adults and the small sample size of this study. Autopsy studies of patients who died from severe COVID-19 have shown diffuse alveolar injury consistent with ARDS and high pulmonary microvascular thrombosis burden (23). High circulating fibrinogen levels indicate hyperinflammatory syndrome (HIS) and hypercoagulable disorder, which are closely associated with adverse outcomes of COVID-19 pneumonia (24). Although the level of D-dimer was not statistically different between the two groups, the higher level of fibrinogen in the pneumonia group suggests that coagulation activation may be involved in the onset of pneumonia. From this perspective, prophylactic anticoagulation may benefit patients with COVID-19 pneumonia.

Domi et al. included 97 adult patients who required mechanical ventilation for severe COVID-19 pneumonia and found that the patients with favorable outcome had increased serum SARS-CoV-2 antibody levels on admission (22). Several randomized clinical trials of COVID-19 convalescent plasma for hospitalized adults with pneumonia have reported that selective participants who received early treatment (60% were SARS-CoV-2 antibody seronegative and had a median of 3 comorbidities) achieved significant benefits in clinical severity scores and 28-day mortality (25), while non-selective participants did not (26). These results suggest that the level of SARS-CoV-2 antibody on admission is closely related to the onset of COVID-19 pneumonia. We also observed consistent results that SARS-CoV-2 IgG titers were 25-fold higher in the non-pneumonia group than the pneumonia group. A recent study showed that vaccinated patients with SARS-COV-2 breakthrough infection showed fewer chest CT findings of pneumonia than unvaccinated patients. However, variant types and specific SARS-COV-2 antibody levels were unknown in this study (21). Comprehensively, boosting vaccination to increase SARS-CoV-2 IgG level may be an important and effective approach for prevention and treatment of Delta variant-induced pneumonia.

In our study, unenhanced axial CT imaging showed that the pneumonia lesions were typically manifested as consolidation and GGO, and predominantly involving both lower lobes with subpleural distribution, and with bronchial wall thickening in a few cases. The predominant pattern of Delta variant-induced pneumonia on CT images could be classified as typical appearance with reference to the proposed reporting language for CT findings related to COVID-19 in the RSNA Expert Consensus Statement (23, 27). The pneumonia lesion occurred at a median of 3 days after admission, peaked within 3 to 7 days after onset, and then began to be absorbed. Compared with the Omicron subvariant BA.2 previously studied in children (15), we found that the Delta variant had more extensive lung involvement and caused more extensive parenchymal changes on chest CT images, which was basically consistent with other emerging studies (5, 18, 19). However, CT images of pneumonia lesions in Delta and Omicron appear to be milder and less recognizable than those in WT and Alpha (17). With the emergence of novel SARS-CoV-2 variants, COVID-19 pneumonia lesions progress rapidly and are not easily identified manually, requiring multiple chest CT scans with a large number of images, which brings a huge workload for radiologists. The image data processing and analysis system based on AI can provide multi-dimensional quantitative information and make image diagnosis automatically, quickly and objectively (4, 15). AI makes it possible to repeatedly measure and quantify pneumonia lesions for longitudinal and interindividual comparisons under different conditions. Therefore, CT image diagnosis system based on AI may have promising applications in the surveillance and diagnosis of novel SARS-CoV-2 variant-induced pneumonia.

Ben-Eltriki et al. found in a meta-analysis that low vitamin D status was statistically associated with a higher risk of death or severe COVID-19 pneumonia (28). Surprisingly, serum 25(OH)D levels were not statistically different between the pneumonia and non-pneumonia groups, suggesting that vitamin D status may not be involved in the onset of Delta variant-induced pneumonia. However, after the onset of pneumonia, patients in the VDD group had higher percentage of fever (33 vs. 7.1%) than those in the control group; the interval of pneumonia resolution was longer (28 vs. 21 days); lesions progressed more rapidly within 3 to 7 days and improved more slowly within more than 28 days; interestingly, simultaneous interleukin-6 (IL-6) levels were higher, and cycle thresholds for N gene and ORF1ab gene were lower within 3 to 7 days; but not for fibrinogen and SARS-CoV-2 IgG. These results are basically consistent with previous studies (13, 15, 24, 28), suggesting that non-VDD status may play a lung protective role by reducing viral load and regulating inflammatory response to inhibit the progression of pneumonia.

It has been demonstrated that enzyme 25(OH)D 1-α hydroxylase (CYP27B1) is upregulated in activated immune cells, so circulating 25(OH)D levels could be lowered by the COVID-19-associated systemic inflammatory response (29). Our study found a significant decrease in serum 25(OH)D levels in the pneumonia group within 3–4 weeks during hospitalization compared to those in the non-pneumonia group and at admission, further providing evidence that detection of vitamin D status and supplementation to correct VDD after Delta variant infection may be justified in preventing severe COVID-19 pneumonia and ARDS (Supplementary Table S4; Supplementary Figure S1). Despite complex mechanisms, 1,25(OH)_2_D (calcitriol) as an active form of vitamin D has been reported to bind to vitamin D receptor (VDR) to exert anti-inflammatory, suppressor cytokine storm effects (13). Calcitriol is currently approved for clinical use as an active vitamin D hydroxymetabolite that works directly without the need for hydroxylation in the liver and kidney and may be very promising. Future expansion on this work should consider measuring and supplementing calcitriol.

Our study have several limitations that warrant mention. (1) Due to the single-center, small sample size, observational nature, there are certain confounding factors. (2) Due to particularly strict quarantine in China, some asymptomatic or mildly symptomatic patients were admitted to hospital in the early stages of COVID-19. The median (IQR) interval from admission to pneumonia onset was 3 (0.25–5) days. The multivariate binary logistic regression analysis may have introduced bias in predicting the onset of pneumonia based on laboratory results in the first day after admission. (3) Our study focused on lesion volume dynamics when evaluating pneumonia with CT imaging, and it is uncertain whether there is a linear correlation between lesion volume and disease severity and prognosis. Consequently, these results should be carefully interpreted and applied clinically due to potential selection bias and residual confounding. Notwithstanding these limitations, our study provides comparative clinical characteristics and CT imaging dynamics of Delta variant-induced COVID-19 pneumonia, which have not been clearly described in the literature.

## Conclusion

Vitamin D status may have effects on the progression and resolution, but not the onset of Delta variant-induced pneumonia in adults. Non-VDD status may play a lung protective role by reducing viral load and regulating inflammatory response to inhibit the progression of pneumonia. CT image diagnosis system based on AI may have promising applications in the surveillance and diagnosis of novel SARS-CoV-2 variant-induced pneumonia.

## Data availability statement

The original contributions presented in the study are included in the article/Supplementary material, further inquiries can be directed to the corresponding author.

## Ethics statement

This investigation involving human participants were reviewed and approved by the Ethics Committee of The Third People’s Hospital of Shenzhen (approval number: 2022-123). Written informed consent for participation was not required for this study in accordance with the national legislation and the institutional requirements.

## Author contributions

HH was responsible for methodology, investigation, formal analysis, data curation, writing the original draft, and visualization. JZ and QZ for investigation and data curation. DP for conceptualization, investigation, review and editing, and supervision. YL worked on conceptualization, formal analysis, investigation, and data curation. All authors contributed to the article and approved the submitted version.

## Funding

This study was supported by the Shenzhen Longgang District Science and Technology Development Fund (grant number: LGKCXGZX2020002).

## Conflict of interest

The authors declare that the research was conducted in the absence of any commercial or financial relationships that could be construed as a potential conflict of interest.

## Publisher’s note

All claims expressed in this article are solely those of the authors and do not necessarily represent those of their affiliated organizations, or those of the publisher, the editors and the reviewers. Any product that may be evaluated in this article, or claim that may be made by its manufacturer, is not guaranteed or endorsed by the publisher.
