# Supplementary material for: Effect of vitamin D status on adult COVID-19 pneumonia induced by Delta variant: A longitudinal, real-world cohort study
Source: Front Med (Lausanne). 2023 Mar 24;10:1121256. doi: 10.3389/fmed.2023.1121256 (PMC10080157; doi:10.3389/fmed.2023.1121256)
Supplement: Supplementary file 1 [file Data_Sheet_1.pdf]

Table S1: Dynamics of the normalized lesion volume of COVID-19 pneumonia between the control and VDD groups

Time period (days)	Control (n=28)	VDD (n=18)	*P* value
$T_1 \leq 3$, Median (IQR)	0.95 (0.29-2.02) n=27	0.35 (0.1-0.79) n=16	0.08
$3 < T_2 \leq 7$	0.71 (0.22-2.93) n=25	2 (0.44-2.95) n=16	0.51
$7 < T_3 \leq 14$	1.47 (0.14-2.44) n=22	1.01 (0.17-2.25) n=16	0.72
$14 < T_4 \leq 21$	1 (0.12-1.66) n=16	0.2 (0.16-1.21) n=13	0.46
$21 < T_5 \leq 28$	0.49 (0.13-0.92) n=15	0.33 (0.03-1.3) n=10	0.87
$T_6 > 28$	0.06 (0.02-0.13) n=9	0.59 (0.09-0.74) n=9	0.04

Abbreviations: COVID-19, coronavirus disease 2019; IQR, interquartile range; VDD, vitamin D deficiency.

Table S2: Progression and resolution of COVID-19 pneumonia lesions between the control and VDD groups

T_{x+1} to T_x ratio	Control (n=28)	VDD (n=18)	*P* value
T_2 to T_1, Median (IQR)	1.05 (0.63-1.78) n=25	2.59 (0.79-9.23) n=16	0.01
T_3 to T_2	0.7 (0.57-1.04) n=21	0.69 (0.28-5.85) n=16	0.44
T_4 to T_3	0.7 (0.42-4.84) n=16	0.65 (0.28-4.45) n=13	0.86
T_5 to T_4	0.71 (0.46-3.36) n=12	0.35 (0.14-2.41) n=9	0.26
T_6 to T_5	0.16 (0.11-1.82) n=7	0.96 (0.63-2.6) n=9	0.007

Abbreviations: COVID-19, coronavirus disease 2019; IQR, interquartile range; VDD, vitamin D deficiency.

Table S3: Effects of vitamin D status on simultaneous laboratory results in patients with Delta variant-induced pneumonia

Time period (days)	Lymphocyte (×10^9)		*P* value
	Control	VDD	
T_0	1.02 (0.69-1.62) n=28	0.82 (0.62-1.04) n=18	0.42
$T_1 \leq 3$	1.42 (1.1-1.74) n=25	1.57 (1.17-1.7) n=15	0.77
$3 < T_2 \leq 7$	1.62 (1.48-2.03) n=23	1.52 (1.38-1.93) n=14	0.57
$7 < T_3 \leq 14$	1.98 (1.72-2.34) n=25	1.73 (1.46-2.35) n=16	0.29
$14 < T_4 \leq 21$	1.89 (1.61-2.11) n=18	1.93 (1.63-2.1) n=14	0.95
$21 < T_5 \leq 28$	1.97 (1.76-2.46) n=16	1.81 (1.57-2.21) n=9	0.24

Time period (days)	Fibrinogen (g/L)		*P* value
	Control	VDD	
T_0	3.5 ± 0.7 n=28	3.5 ± 1 n=18	0.25
$T_1 \leq 3$	3.8 ± 0.7 n=25	3.6 ± 0.6 n=13	0.51
$3 < T_2 \leq 7$	4 ± 0.9 n=23	3.8 ± 0.8 n=13	0.53
$7 < T_3 \leq 14$	3.7 ± 0.8 n=24	3.3 ± 1 n=15	0.09
$14 < T_4 \leq 21$	3.3 ± 0.6 n=17	3.1 ± 0.6 n=12	0.34
$21 < T_5 \leq 28$	3 ± 1 n=16	2.9 ± 0.3 n=8	0.36

Time period (days)	D-dimer (μg/mL)		*P* value
	Control	VDD	
T_0	0.25 (0.22-0.35) n=28	0.24 (0.22-0.3) n=18	0.41
$T_1 \leq 3$	0.37 (0.23-0.46) n=25	0.3 (0.22-0.34) n=13	0.13
$3 < T_2 \leq 7$	0.39 (0.28-0.49) n=22	0.37 (0.22-0.44) n=13	0.24
$7 < T_3 \leq 14$	0.41 (0.23-0.47) n=24	0.28 (0.23-0.34) n=15	0.19
$14 < T_4 \leq 21$	0.26 (0.22-0.41) n=17	0.22 (0.22-0.33) n=12	0.35
$21 < T_5 \leq 28$	0.24 (0.22-0.38) n=14	0.23 (0.22-0.29) n=8	0.77

Time period (days)	Procalcitonin (ng/mL)		*P* value
	Control	VDD	
T_0	0.058 (0.034-0.074) n=28	0.044 (0.04-0.064) n=18	0.59
$T_1 \leq 3$	0.052 (0.044-0.062) n=25	0.049 (0.042-0.065) n=15	0.97
$3 < T_2 \leq 7$	0.055 (0.043-0.067) n=22	0.047 (0.04-0.055) n=14	0.28
$7 < T_3 \leq 14$	0.044 (0.04-0.052) n=25	0.044 (0.031-0.077) n=16	1.00
$14 < T_4 \leq 21$	0.044 (0.037-0.061) n=18	0.039 (0.036-0.049) n=13	0.31
$21 < T_5 \leq 28$	0.051 (0.042-0.059) n=14	0.045 (0.031-0.059) n=9	0.64

Time period (days)	IL-6 (pg/mL)		*P* value
	Control	VDD	
T_0	13.9 ± 6 n=26	14.4 ± 4.8 n=18	0.74
$T_1 \leq 3$	14.6 ± 6.2 n=25	18.7 ± 5.5 n=15	0.04
$3 < T_2 \leq 7$	17.4 ± 8 n=22	16 ± 3.4 n=13	0.56
$7 < T_3 \leq 14$	12.6 ± 5.2 n=26	15.7 ± 7.2 n=16	0.11
$14 < T_4 \leq 21$	11.8 ± 3.3 n=17	11.9 ± 4 n=13	0.97
$21 < T_5 \leq 28$	9.8 ± 4.9 n=14	12.2 ± 3.4 n=8	0.25

Time period (days)	SARS-CoV-2 IgM titers		*P* value
	Control	VDD	
T_0	0.1 (0.05-0.29) n=28	0.19 (0.08-0.45) n=18	0.18
$T_1 \leq 3$	0.58 (0.23-4.03) n=19	1.32 (0.18-3.23) n=13	0.83
$3 < T_2 \leq 7$	5.42 (2.41-12.12) n=20	3.35 (0.49-6.05) n=12	0.09
$7 < T_3 \leq 14$	5.63 (3.03-16.66) n=21	4.22 (0.68-19.13) n=12	0.65
$14 < T_4 \leq 21$	5.16 (3.16-7.69) n=14	2.36 (0.81-11.35) n=12	0.36
$21 < T_5 \leq 28$	4.77 (2.39-10.82) n=14	12.96 (4.26-18.41) n=9	0.15

Time period (days)	SARS-CoV-2 IgG titers		*P* value
	Control	VDD	
T_0	4.4 (0.6-20.5) n=28	3.5 (0.6-80.1) n=18	0.99
$T_1 \leq 3$	150.6 (28.8-256.7) n=19	213.8 (6.6-350.1) n=13	0.74
$3 < T_2 \leq 7$	340.3 (242.5-416.5) n=20	328.2 (131-408.1) n=12	0.53
$7 < T_3 \leq 14$	371 (306.1-396.1) n=21	346.8 (148.9-401.2) n=12	0.48

14 < T_4 ≤ 21	375.9 (298.6-412.5) n=14	385.7 (354.5-393.5) n=12	0.96
21 < T_5 ≤ 28	355.9 (301.6-399.3) n=14	359.1 (324-397.5) n=9	0.85

Time period (days)	Cycle threshold for N gene		*P* value
	Control	VDD	
T_0	26.2 ± 8.5 n=17	23.7 ± 8 n=10	0.45
T_1 ≤ 3	31.3 ± 8 n=12	22.8 ± 7.9 n=7	0.04
3 < T_2 ≤ 7	31.5 ± 7.7 n=18	30.2 ± 6.9 n=7	0.69
7 < T_3 ≤ 14	29.6 ± 7.3 n=16	30.9 ± 7.8 n=7	0.73
14 < T_4 ≤ 21	30.9 ± 6.9 n=10	31.7 ± 7.7 n=6	0.85
21 < T_5 ≤ 28	30.7 ± 7.3 n=12	30.2 ± 7.4 n=6	0.88

Time period (days)	Cycle threshold for ORF1ab gene		*P* value
	Control	VDD	
T_0	25 ± 8 n=15	20.8 ± 3.7 n=8	0.17
T_1 ≤ 3	28.7 ± 6.9 n=8	20.9 ± 4.2 n=6	0.03
3 < T_2 ≤ 7	29.2 ± 8.2 n=12	26.5 ± 7.4 n=5	0.53
7 < T_3 ≤ 14	29 ± 7.8 n=14	21.7 ± 5.3 n=5	0.15
14 < T_4 ≤ 21	30 ± 6.6 n=8	32.9 ± 6.9 n=6	0.49
21 < T_5 ≤ 28	30.1 ± 7.4 n=9	29.9 ± 8.7 n=5	0.97

Abbreviations: IL-6, interleukin-6; IQR, interquartile range; SARS-CoV-2, severe acute respiratory syndrome coronavirus 2; SD, standard deviation; VDD, vitamin D deficiency.

Table S4: Serum 25(OH)D dynamics in patients infected with the Delta variant

Time period (days)	Serum 25(OH)D concentration (ng/mL)		*P* value
	Non-pneumonia (n=115)	Pneumonia (n=46)	
T_0	21 (16.9-25) n=115	22.3 (17.2-28.6) n=46	0.28
T_1 ≤ 3	20.6 (16.7-24.5) n=51	22.8 (17.9-28.3) n=32	0.15
3 < T_2 ≤ 7	18.1 (16.2-22.4) n=36	20.6 (17.5-26.1) n=26	0.13
7 < T_3 ≤ 14	20.2 (17.5-25.3) n=35	18.2 (15.3-21.6) n=25	0.18
14 < T_4 ≤ 21	18.9 (15.9-22.6) n=40	19.3 (16.3-23.6) n=20	0.27
21 < T_5 ≤ 28	20.1 (17.9-23.2) n=28	16.2 (15.9-18.7) n=5	0.02

Time period (days)	Serum 25(OH)D concentration (ng/mL)		*P* value
	Control (n=28)	VDD (n=18)	
T_0	27.1 ± 4.4 n=28	16.3 ± 2.2 n=18	<0.001
T_1 ≤ 3	27.7 ± 4.9 n=19	17.4 ± 2.1 n=13	<0.001
3 < T_2 ≤ 7	24.6 ± 5.1 n=16	17.1 ± 2.9 n=10	<0.001
7 < T_3 ≤ 14	22.2 ± 4.2 n=14	14.6 ± 2.8 n=11	<0.001
14 < T_4 ≤ 21	24.4 ± 5.1 n=12	15.8 ± 1.3 n=8	<0.001
21 < T_5 ≤ 28	16.7 ± 2 n=2	17 ± 2 n=3	0.89

Figure S1

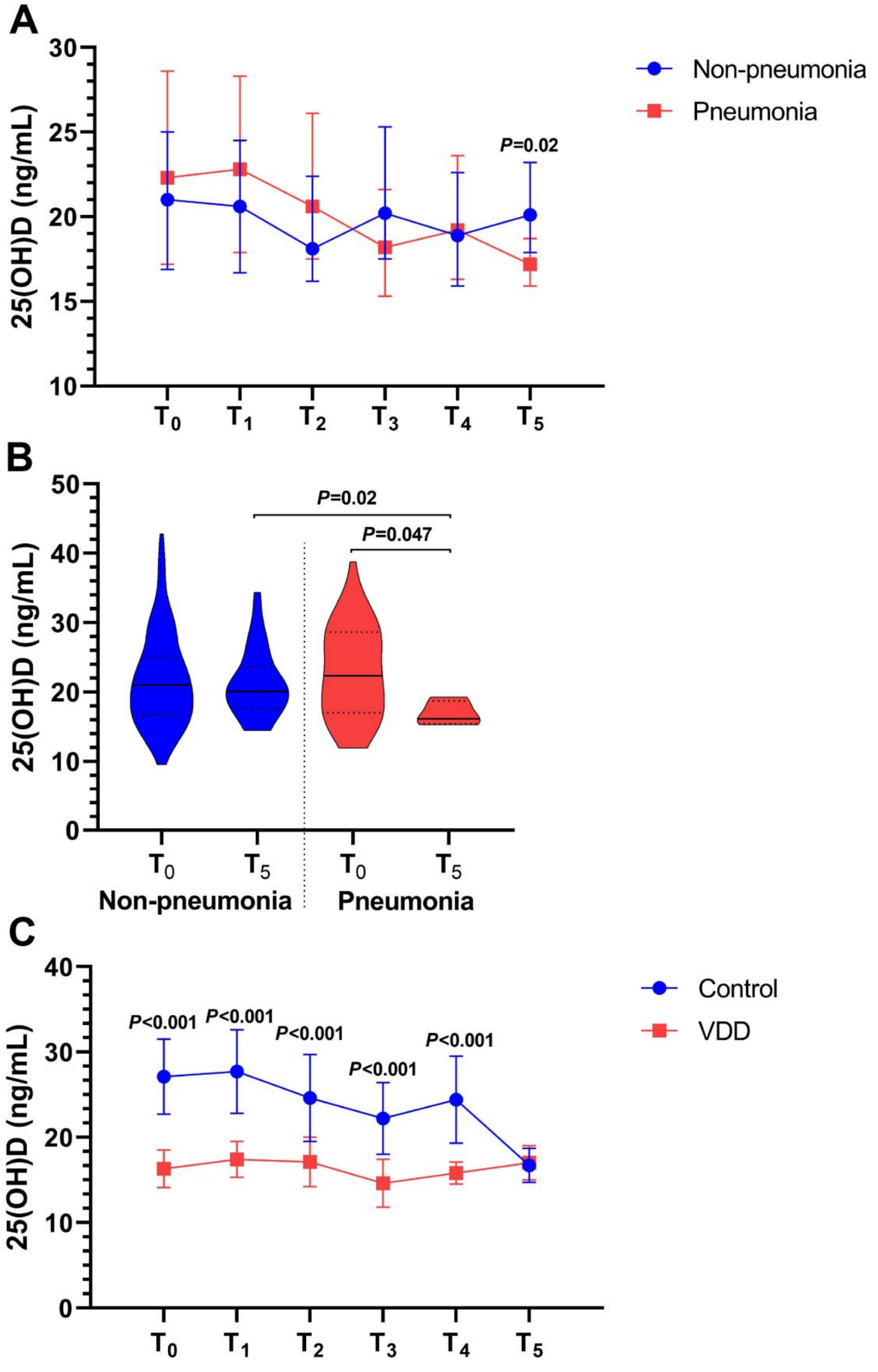
A
25(OH)D (ng/mL)
Non-pneumonia
Pneumonia
P=0.02
T_0 T_1 T_2 T_3 T_4 T_5
B
P=0.02
P=0.047
T_0 T_5 T_0 T_5
Non-pneumonia
Pneumonia
C
Control
VDD
P<0.001
P<0.001
P<0.001
P<0.001
P<0.001
